# The prevalence of malaria among HIV seropositive individuals and the impact of the co- infection on their hemoglobin levels

**DOI:** 10.1186/s12941-015-0064-6

**Published:** 2015-03-07

**Authors:** Sammy CK Tay, Kingsley Badu, Anthony A Mensah, Stephen Y Gbedema

**Affiliations:** Department of Clinical Microbiology, School of Medical Sciences, Kwame Nkrumah University of Science and Technology, Kumasi, Ghana; Department of Immunology, Noguchi Memorial Institute for Medical Research, College of Health Science, University of Ghana, Accra, Ghana; Department of Microbiology, Holy Family Hospital Techiman, Techiman, Ghana; Department of Pharmaceutics, Faculty of Pharmacy and Pharmaceutical Sciences, College of Health Sciences, Kwame Nkrumah University of Science and Technology, Kumasi, Ghana

**Keywords:** HIV/AIDS, CD_4_ cells, Malaria co-infection, Anaemia, Anti-retroviral

## Abstract

**Background:**

Malaria and HIV/AIDS are the two most common infections in sub-Sahara Africa. There are hypotheses and study reports on the possible association between these two infections, hence the prevalence and outcome of their co-infection in an endemic population will be important in defining healthcare strategies. A cross sectional study was carried out at the Holy Family Hospital in Techiman, Ghana, between November 2011 and January 2012, to determine the prevalence of malaria among HIV sero-positive patients and its impact on hemoglobin levels.

**Method:**

A total of 400 HIV sero-positive participants (292 females and 108 males) aged between 1 and 73 years were randomly sampled for the study. A questionnaire was administered and 2 ml of venous blood samples were drawn for malaria parasites detection, CD4 count and haemoglobin level estimations.

**Results:**

Malaria parasites were detected in 47 (11.75%) of the participants. There was no statistically significant difference between the malaria prevalence rate of females (12.1%) and males (10.2%) P = 0.6047. An overall anaemia prevalence of 67% was observed. Among participants with malaria the anaemia prevalence was 93.6%. The CD4 cell count of all the participants ranged between 3 and 1604 cells/μl with a mean of 386.2 (±274.3) cells/μl. Participants with malaria had CD4 cell count ranged 3 and 512 Cells/μl with the mean being 186.33 (±133.49) Cells/μl. Out of 377 participants (all above 15 years) interviewed on knowledge of malaria transmission and prevention, 87.0% had knowledge on transmission but only 8.5% use in bed nets.

**Conclusion:**

It was revealed that almost all the patients with malaria infection were anemic.

## Introduction

Malaria and Human immunodeficiency virus (HIV) infections are major public health problems in many parts of the world. Both infections kill millions of people each year with disproportionate heavy burden on Africa, India, Southeast Asia and South America [1].

Sub-Saharan Africa harbors 90% of the estimated 219 million cases that results in over 600,000 deaths annually [2,3]. Severe anemia as a result of malaria occurs in 1.5 to 6.0 million African children annually with mortality rate of nearly 10%; respiratory distress, hypoglycaemia and other complications result in an additional 1–2 million cases [4]. Malaria is a major cause of maternal anemia, which in turn is a risk factor for maternal mortality resulting in about 35% of preventable cases of low birth weight [4]. In Ghana malaria is still the leading cause of loss of days of healthy life [5] as it accounts for at least 20% of child deaths and over 45% of out-patient attendances [5].

Because of the high prevalence of HIV and malaria in sub-Saharan Africa, co-infections are common. Recent estimates put HIV/AIDS prevalence in Ghana at 2.9% [6]. It is known that some 225,478 persons are living with the disease. Further to this, about 12,077 new infections are recorded annually with corresponding 15, 263 deaths [6,7]. By 2011, 59,007 people had access to anti-retroviral treatment; however this represented 57.9% of eligible patients [6]. Implying that an excess of 40% of people living with HIV/AIDS are left to die without getting access to anti-retroviral treatment. Notwithstanding the public huge health burden presented by these two infections, their interaction is still not completely understood [1].

Because of the high prevalence of HIV and malaria in sub-Saharan Africa, co-infections are common, Because of the geographic overlap between high-prevalence areas for malaria and HIV infection, there is growing evidence that the two infections may synergistically intensify each other, increasing incidence and complicating treatment efforts [3]. In a region of unstable malaria, HIV infection had an unexpectedly large association with the outcome of *falciparum* malaria where HIV infection was associated with severe/complicated malaria [8]. Recently Gupta and Shah using Sensitivity analysis and simulation has shown that malaria makes people move faster from HIV to AIDS class and reduce their life span. Radad and coworkers reported that an increase of one log in HIV viral load occurs during febrile malaria episodes enhancing susceptibility to malaria in HIV infected patients and this was found to facilitate the geographic expansion of malaria in areas where HIV prevalence was high [9].

As the number of malaria and HIV co-infection increased, it has become apparent that anti-retroviral drugs interact with the few anti-malaria drugs in use, complicating treatment efforts for both infections [10]. Malaria and HIV co-infection also result in interactions that adversely affect the outcome of both conditions, especially among pregnant women and infants born to HIV infected mothers [11]. Malaria [12] and HIV [13] individually are known to cause maternal anemia. Further to this, a number of researchers have reported a negative effect of the combined impact of HIV and malaria on maternal hemoglobin (Hb) concentrations [14]. However, there is a dearth of information on the collective impact of HIV-malaria co-infection on the hemoglobin levels in the general population. Understanding the impact of HIV and malaria co-infection therefore, is important for determining approaches to treatment and prevention.

## Methods

The study was conducted in the Holy Family Hospital; the only facility that provides ART services to HIV patients in the municipality. It has bed capacity of 167 beds. Patients diagnosed to have HIV/AIDS infection and who sought treatment at ART clinic at the Holy Family Hospital were the source population for the study. The drugs administered for the first line of treatment were Azidovudine (d4T), Lamivudine (3TC) plus Nevirapine or Efavirenz. Nelfinavir, Kaletra and Indinavir were occasionally used in combination with nucleoside based reverse transcriptase inhibitor drugs. The study was cross-sectional serological survey where HIV sero-positive patients attending ART Clinic at the hospital were randomly approached and invited to participate in the study provided they gave their consent. The study took place between November 2011 and January 2012.

Sample size was determined using the binomial model to estimate the confidence interval (CI). The HIV prevalence in the area is known to be less than 3% so the malaria prevalence in the area which is higher was used to estimate the minimum sample size needed to achieve enough statistical power. Malaria prevalence in the area had previously been determined by Owusu-Adjei et al. to be about 60% in all age cohorts [15]. We calculated the sample size with a 95% CI and precision level of 5%:

$$ {n}_o=\frac{Z_aPq}{d^2} $$

In the equation below, *n* is the sample size, *z* is the critical value of the standard normal distribution at the 5% level (1.96), p is the estimated malaria prevalence (0.60), *q* = 1 – *p*, and *d* is the precision level. This is used for a small population of up to 3,000. The sample size obtained was 369. A total of 400 HIV sero-positive individuals who sought care at the ART Clinic of the Holy Family Hospital in Techiman and were willing to participate in the study were recruited.

### Study area

Techiman, is a major commercial town in the Brong Ahafo region linking not only most of Ghana’s major cities, but also the republic of Togo, Burkina Faso and Cote D’Ivoire. It has an estimated total land area of about 669.7 square kilometers. According to the 2000 population and housing census, the population of the municipality was estimated to be 202,409 by December 2005, with an average growth rate of 3.0% per annum. Agriculture and related trades are the major economic activities in the municipality. Over half of the economically active population is engaged in these activities. This makes Techiman market the largest food crop market in Ghana. The municipality has twenty four (24) health care facilities; including two mission hospitals in Techiman, (i.e. Holy Family and Ahamadiya Hospitals), nine government health centers, four private maternity homes and three private clinics.

### Ethical clearance

The study was approved by the Committee on Human Research Publication and Ethics from Kwame Nkrumah University of Science Technology and the management of Holy Family Hospital - Techiman. Consent of participants was also obtained prior to the study. All consenting patients attending the ART clinic were eligible for participation except infants less than 6 months. Refusal of some patients to participate in the study did not interfere with their routine care at the hospital.

### Structured questionnaire

A questionnaire, in both English and *Twi* (the local language) which included the basic socio- demographics of the study subjects as well as their knowledge and prevention of malaria was used. Two independent translators were engaged, one translated the English to *Twi,* the other was who had no prior copy of the English was tasked to translate the local language back into English. This was done to ensure the consistency of thought. No personal identifiers were included and individuals were given unique codes on the questionnaires and their laboratory specimens.

### Malaria parasite

Laboratory investigation was conducted using the Thin Blood Film method. A drop of blood, 3–5 mm in diameter was put on a slide and spread with the corner of another slide to form a thin blood smear. The smear was thoroughly allowed to dry, fixed with methanol and stained with Giemsa stain for about 15 minutes. The stained smear was washed with running tap water, allowed to dry in air and examined microscopically for the presence of malaria parasites.

### HIV infection

HIV test was conducted using First Response HIV Card Test 1–2.0 (Premier Medical Corporation Ltd, Mumbai, India). Reactive blood samples were confirmed with Oraquick Rapid HIV – ½ Antibody Test (OraSure Technologies Inc., Bethlehem, USA). Both test kits are immuno-chromatographic rapid tests for qualitative detection of antibodies specific to HIV in human serum, plasma or whole blood.

### CD4 count and Haemoglobin concentration

The CD4 count of the study population was determined using Becton-Dickinson FASCount flow cytometer (BD Biosciences, California, USA) whilst the haemoglobin concentration was estimated with Sysmex XT – 2000i Haematology Analyzer (Sysmex Corporation, Hong Kong, China).

## Results

### Socio-economic and demographic characteristics

A total of 400 HIV sero-positive patients between 1 and 73 years were included in the study. Majority of them (73%). the mean was between 30 and 34 years old. Most patients were married (38%). Majority (37.1%) had no formal education, with only 3% having had some tertiary education. 49.5% of the participants were unemployed with a further 30.7% being farmers. Further details are reported in Table 1.

**Table 1:** **Socio-economic and demographic characteristics of patients (participants)**

**Gender**	**Frequency**	**Percentage (%)**
Male	108	27.0
Female	292	73.0
**Age (years)**		
1-4	12	3.0
5 -9	2	0.5
10 -14	9	2.3
15 – 24	37	9.3
25 – 34	147	36.8
35- 44	118	29.5
45 – 60	69	17.5
>60	5	1.25
**Marital status**		
Married	152	38%
Single	140	35%
Divorced/Widowed	108	27%
**Education**		
No formal education	151	49.5
Primary education	98	24.5
JHS/O-Level	103	25.8
SHS/A-Level	36	9.0
Tertiary	12	3.0
**Occupation**		
Unemployed	198	49.5
Farmers	123	30.8
Traders	49	12.3
Other Skills	30	7.5

### Hemoglobin level of patients (participants)

**Figure 1:**
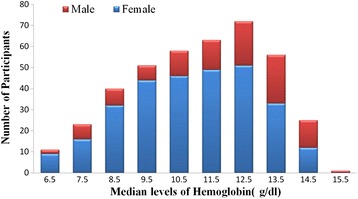
**Median hemoglobin levels of participants in different gender.**

Hemoglobin (Hb) concentration levels of the patients ranged between 6.4 and 15.4 g/dl with majority (18.0%) of patients had Hb levels in the range of 12.-12.9 g/dl. The mean Hb level of the patients was 10.8 g/dl with mean (SD) for male and female as 11.5 g/dl (2.26) and 10.56 g/dl (2.22) respectively (Table 1, Figure 1). Per the definition of anemia by the World Health Organization, hemoglobin levels < 13.0 g/dl in adult men than < 12.0 g/dl in adult women, an overall prevalence of 67% (268 out of 400) anemia was observed in this study. Majority of patients had mild to moderate anemia while severe anemia was observed in 2.8% of patients. Further details are reported in Figure 1 and Table 2.

**Table 2:** **Hemoglobin levels in different gender**

**Hemoglobin (g/dl)**	**Male frequency (%)**	**Female frequency (%)**	**Total frequency (%)**
**6-6.9**	2 (1.9)	9 (3.1)	11 (2.75)
**7-7.9**	7 (6.5)	16 (5.4)	23 (5.75)
**8-8.9**	8 (7.4)	32 (11.0)	40 (10.0)
**9-9.9 (12.75)**	7 (6.5)	44 (15.1)	51
**10-10.9 (14.50)**	12 (11.1)	46 (15.8)	58
**11-11.9 (15.75)**	14 (13.0)	49 (16.8)	63
**12-12.9 (18.00)**	21 (19.4)	51 (17.4)	72
**13-13.9 (14.00)**	23 (21.3)	33 (11.3)	56
**14-14.9 (6.25)**	13 (12.0)	12 (4.1)	25
**15-15.9**	1 (0.9)	0 (0.00)	1 (0.25)

### CD4 count of patients

All patients studied had a CD4 cell count ranging from 3 – 1604 cells/μl. The mean (SD) of CD4 cell count of the patients was 386.2 (274.3) cells/μl. Majority of the participants (34.5%) had CD4 count below 200 cells/μl (this represents the advanced stage of immuno-suppression). Another 33.3% had CD4 counts above 500cells/μl. The rest of the participants had CD4 counts between 200 and 499 cells/μl. further details of CD4 cell count based on gender differences are shown in the tables (Table 3).

**Table 3:** **CD4 count of participants stratified according to gender**

**CD4 count**	**Male frequency (%)**	**Female frequency (%)**	**Total frequency (%)**
<200	46 (42.6)	92 (31.5)	138 (34.50)
200-349	23 (21.3)	54(18.5)	77 (19.3)
350-499	12 (11.1)	40 (13.7)	52 (13.0)
≥500	27 (25.0)	106 (36.3)	133(33.3)

### Malaria parasite prevalence

**Figure 2:**
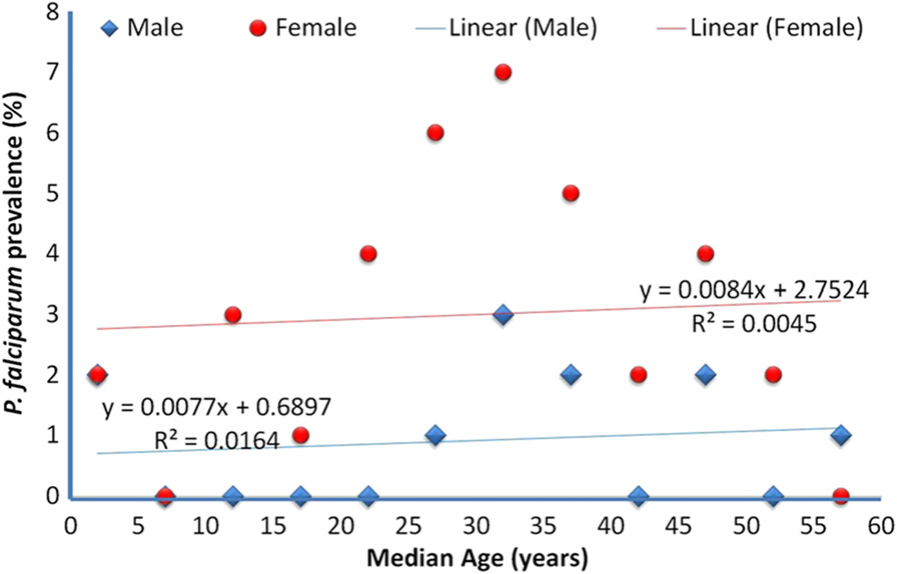
**Age distribution of patients with malaria infection.**

An overall 11.75% (n = 47) were found to be slide positive for malaria parasite with ages ranging from 2 years to 56 years. Malaria positivity was mostly within the age range of 30–34 years, followed by those in the age ranges of 25–29 years and 35–39 years. The least infected malaria patients were within the age ranges of 15-19years and 55–59 years. Out of the 47 participants with patent parasitemia 36 (76%) of them were females with only 11 being males (Figure 2).

### Malaria co infected participants and hemoglobin levels

**Figure 3:**
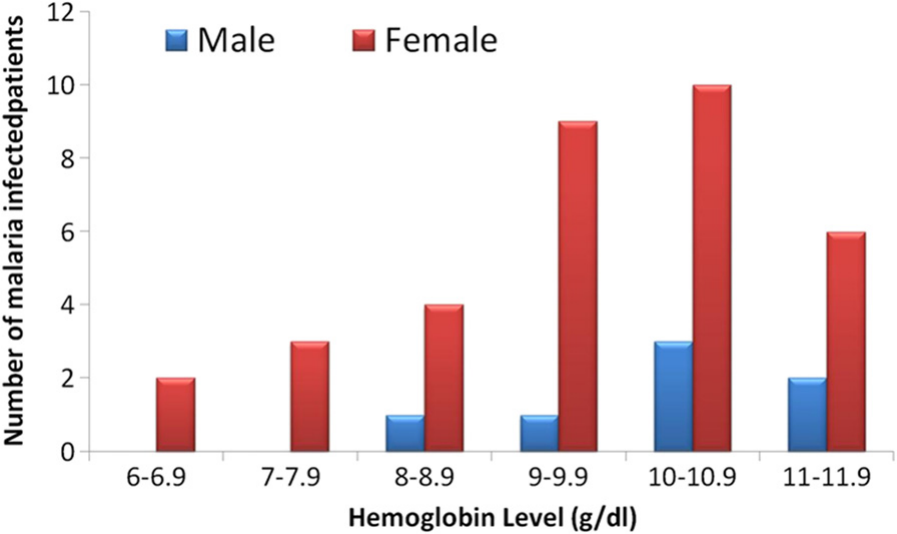
**Hemoglobin level HIV patients Co-infected with malaria stratified according to gender.**

All participants who had malaria infection had a mean (SD) haemoglobin level to be 9.9 g/dl (1.61). The hemoglobin level ranged was from 6.2 g/dl to 13.2 g/dl. All the participants with malaria infection were anemic except for one participant who had 13.2 g/dl. Most of the study participants with malaria infection (13 out of 47) had hemoglobin level between the ranges of 10.0 g/dl-10.9 g/dl (Figure 3). The prevalence of anemia with respect to gender was 90.9% and 94.4% for males and females respectively. Majority of the subjects with malaria infection 45 (89.4%) had mild to moderate anemia with only 4.3% (2 out 47) having severe anemia. Severe anemia was observed in only females (Figure 3).

### Malaria infection and CD4 count

**Figure 4:**
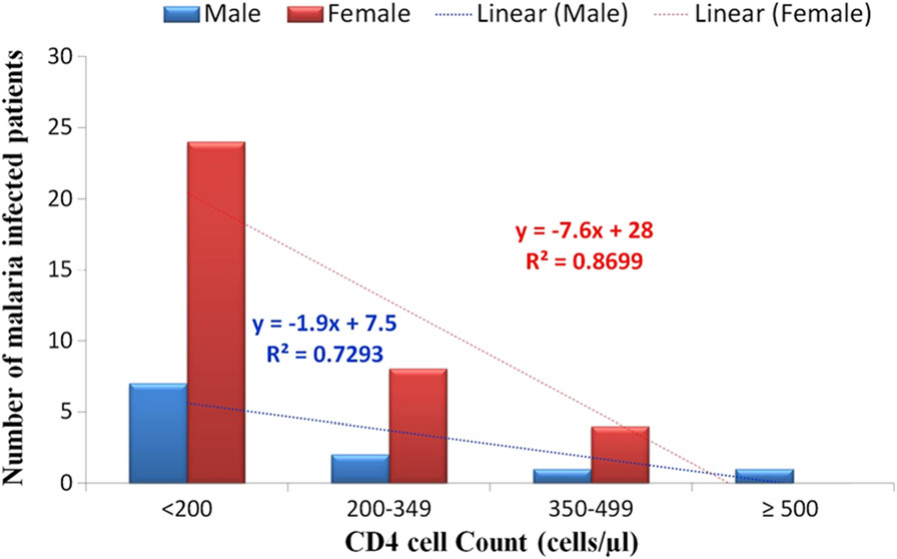
**CD4 count distribution of patients infected with malaria.**

All patients with malaria infection had CD4 cell count ranging from 3 cells/μl to 512 cells/μl with mean (SD) CD4 cell count of 186.3 (133.5) cell/ul. Males had a mean of 209 (198.4) cells/μl and females 178.7 (110.8) cells/μl. The majority, a whopping 66% of participants with patent malaria infection had their CD4 cell count below 200 cells/μl. With respect to gender, 63.6% (7 out of 11) males infected with malaria infection and 66.7% (24 out of 36) females infected with malaria infection had CD4 cell count less than 200 cells/μl. Only one patient with malaria infection had CD4 cell count greater than or equal to 500 cells/μl and was observed in only males. Further details are provided in (Figure 4).

### Knowledge of malaria transmission and prevention

Participants above 15 years of age (n = 377) interviewed on malaria transmission and prevention. 87.0% out of 377 of the participants claimed to have some knowledge about malaria transmission. Out of the 328 respondents that claimed to have knowledge about malaria, 216 (65.9%) claimed that malaria could be transmitted from an infected person to a healthy person and the rest 112 (34.1%) claimed that either malaria cannot be transmitted from person to person or they had no idea at all. Among those who claimed that malaria is transmissible (n = 216), 91.2% (n = 197) reported mosquito bite as a means of transmission (Table 4). About 92.7% (304) of study participant who claimed to have knowledge about malaria reported that malaria is preventable. The most frequently mentioned method of prevention was environmental sanitation 286 (94.1%) followed by taking antimalarial tablets 238(78.3%), bed nets 231(76.0%) and mosquito repellent 196 (64.5%). The remaining 87(28.6%) reported various other methods like traditional cotton clothes, smoke and good diet. It was however surprising that with all the knowledge they claim to have, only 8.5% (n = 32) participants actually used bed net (Table 5).

**Table 4:** **Knowledge of malaria transmission and prevention**

**Variable**	**No. of respondents**	**Frequency (%)**
**Knowledge about malaria**	(n =377)	
Yes		328 (87.0)
No		49 (13.0)
**Malaria transmissible?**	(n = 328)	
Yes		216 (65.9)
No		112(34.1)
**Means of malaria transmission**	(n =216)	
Mosquito bite		197 (91.2)
Body contact		13 (3.4)
Respiratory route		24 (11.1)
Others		28 (13.0)
**Malaria is preventable**	(n = 328)	
Yes		304 (92.7)
No		24 (7.3)
**Preventive methods**	(n =304)	
Antimalarial tablets		238 (78.3)
Bed nets		231 (76.0)
Mosquito repellents		196 (64.5)
Environmental sanitation		286 (94.1)
Others		87 (28.6)
**Use of bed nets**	(n = 377)	
Yes		32 (8.5)
No		345 (91.5)

**Table 5:** **CD4 cell count in participants co-infected with malaria**

**CD4 Count**	**Frequency**	**Male**	**Female**	**P –value**
<200	31 (63.6)	7(63.6)	24 (66.7)	0.014
200-349	10 (21.3)	2(18.2)	8(22.2)	0.235
350-499	5(10.6)	1(9.1)	4(11.1)	0.580
>500	1 (2.1)	1(9.1)	0(0.0)	1.000

## Discussion

Accumulating data points to pathological interactions between HIV and malaria in co infected individuals. However, the public health consequences of this interaction have not been completely deciphered. Abu-Radad and coworkers in Kenya observed that a temporary one-log elevation in HIV viral load occurs during febrile malaria episodes; in addition, susceptibility to malaria is enhanced in HIV-infected patients [11].

In a one-time cross-sectional serological survey, the current study sought to determine malaria prevalence in HIV seropositive individuals; their CD4 count to identify the various stages of immuno-suppression and their hemoglobin levels to possibly associate the impact of the co-infection on the outcome of anemia in the study participants.

Generally malaria transmission in the forest zone of Ghana is intense [6,15] with very high parasite prevalence up to 50% [15,16]. But the overall malaria prevalence in this study was low; this may be due to the fact that the study was conducted during the drought months (November- January) which coincide in Ghana with very low malaria transmission. However in comparison with similar studies in the African region, malaria prevalence in this study appears to be higher. The prevalence of malaria infection among HIV sero-positive patients in South Africa and Mozambique were lower [17,18]. But the observed prevalence is similar with a study done in Southeastern part of Nigeria in which the prevalence of malaria as a co-infection amongst asymptomatic HIV sero positive patients was 11.8% [19]. Malaria is known to cause an increase in transitory viral load while HIV causes more clinical malaria, higher parasitemia and higher rates of treatment failure in co-infected patients [11].

Females had relatively higher malaria infection compared with their male counterparts. This may be explained by their typical lifestyle in the community, where females tend stay out late during mosquito biting hours carrying out domestic activities. This has variously been reported elsewhere. Also, among the various age groups, it was determined that the age 30–34 years harbored more parasites than the other age groups. This was also confirmed with the high cumulative density index. However, prevalence of malaria infection was high in children within the age group of 0–4 years in which out of 12 patients, four patients representing 33.3% had malaria infection. This is consistent with several studies in malaria endemic Africa [19] where prevalence of malaria infection in HIV children was high. However some studies have reported lower prevalence of malaria among non HIV children less than five years in the Brong-Ahafo region of Ghana [20].

The mean (SD) CD4 cell count of the patients was 386.2 (274.3). Males and females had CD4 count mean (SD) of 345.7(270.1) cells/μl and 386.1(276.0) cells/μl respectively. The difference in the mean CD4 count is due to the fact that HIV infection is detected earlier in females than their male counterparts. With the introduction of Prevention of Mother -To- Child Transmission of HIV (PMTCT) programme, women of child bearing age are screened for HIV infection during antenatal care or at obstetrics and gynecology clinics. Those who test positive are then incorporated into ART clinics while there is no similar program for males for the early detection of HIV.

CD4 cell count less than 200 cells/μl (signifying the terminal stage of HIV infection (AIDS) was seen in 34.5% patients. Obviously the major reason for this high proportion of AIDS is because of late presentation, largely due to the fact that we are yet to imbibe the culture of voluntary screening for early detection and treatment. Fear of stigmatization, lack of awareness and inadequate trained counseling personnel are some of the factors militating against voluntary screening. Many patients only seek medical attention and are diagnosed when HIV infection becomes complicated by AIDS defining illnesses. Majority of the patients (66.0%) with malaria infection had CD4 cells count less than 200 cells/μl. This can be attributed to the well establishment that CD4^+^ T lymphocyte cells <200 cells/μl is associated with a higher risk of opportunistic infection and poor disease progression [16].

Anemia is a frequent complication of infection with the human immunodeficiency virus, and could be clinically important. Multifactorial origin of anemia complicates determining its original cause and/or its proper treatment [21]. Using hemoglobin level of less than 13.0 g/dl and 12.0 g/dl as the cutoff point for anemia in males and females respectively, the overall prevalence of anemia among the patients was 67%. The prevalence of anemia in this study is comparable with other studies (approximately 70%) [22,23]. The incidence of anemia was strongly and consistently associated with the progression of HIV disease as measured by diagnosis of an AIDS-defining opportunistic illness and measurement of a CD4 count of 200 cells/μl. This association is most likely explained by the increasing viral burden as HIV disease progresses, which could cause anemia by increased cytokine mediated myelosuppression which impair erythropoiesis. Also several opportunistic organisms like *Mycobacterium tuberculosis*, *Histoplasma*, *Cryptococcus*, *Coccidiodes*, *Pneumocystis carinii*, and *Leishmania* have been shown to infiltrate the bone marrow and disrupt erythropoiesis [24]. Alternatively anemia may be a surrogate marker for some aspect of disease progression not captured by controlling for CD4 count and clinical AIDS diagnosis.

Several drugs used to combat HIV and its complications may contribute to the anemia that is seen in HIV infection. The administration of Zidovudine is recognized to cause anemia because of myelo-suppression [25].

Males and females did not show significant difference in the prevalence of anemia. Different studies have reported increase prevalence of anemia in females than males which were largely attributed to menstrual blood loss and to the drains on iron stores that occur with pregnancy and delivery [21]. However in this study, it was revealed that most males report to the ART clinic late by which HIV infection without antiretroviral therapy in the vast majority of infected males has progressively destroyed the immune system leading to opportunistic diseases and other condition that lead to anemia in HIV infection has advanced.

Majority of the HIV patients with anemia were mild to moderate anemia with few patients (2.75%) with severe anemia. This is in agreement with studies by Meidani and co workers [21] where mild to moderate anemia was observed in majority of HIV patients with anemia in their studies. It was revealed that almost all the patients with malaria infection were anemic. This may be due to the clearance and or destruction of infected RBCs, the clearance of uninfected RBCs, erythropoietic suppression and dyserythropoiesis as reported as reported by others elsewhere by [26].

About 87% of the patients who responded to the questionnaire claimed to have some knowledge on malaria transmission in their area. This is an indication that the area selected for the study is malarious and known to have malaria transmission. More than half (65.9%) of the respondents who claimed to know or have heard of malaria, reported malaria to be transmissible. The rest (34.1%) reported that malaria cannot be transmitted from infected person to healthy ones or they have no idea about it at all. Among those who knew that malaria is transmitted, 91.2% reported mosquito bite as a means of transmission. These are encouraging results and show the presence of a better knowledge in the method of malaria transmission however despite good knowledge about malaria transmission, this study also revealed evidence of knowledge gaps about malaria by some respondents misconception that that malaria is transmitted through drinking contaminated water, eating contaminated food, staying in the sun and working in rain.

Over 92.0% of the patients in this study reported that malaria is preventable. Over 75% of the patients who reported malaria are preventable stated taking tablets and use of bednets as methods of malaria prevention however only 8.5% use bednets. Discomfort, primarily due to heat, and perceived (low) mosquito density were the most widely identified reason for non-use of mosquito net. There were concerns by respondents about higher prices of mosquito nets to the extent of not being affordable by some respondents.

Malaria infection was high in HIV positive patients with CD4 count less than 200 cells/μl. Mild to moderate anemia was frequent in HIV positive patients but severe anemia was not prevalent in the study population. Almost all study participants with malaria were found to be anemic. This confirms the association between malaria and anemia. Future studies should compare prevalence of malaria co-infection among HIV/AIDS Sero-positive individuals and non HIV/AIDS individuals with malaria.
